# Iontophoresis of Endothelin Receptor Antagonists in Rats and Men

**DOI:** 10.1371/journal.pone.0040792

**Published:** 2012-07-13

**Authors:** Matthieu Roustit, Sophie Blaise, Claire Arnaud, Marcin Hellmann, Claire Millet, Diane Godin-Ribuot, Boris Dufournet, Jean Boutonnat, Christophe Ribuot, Jean-Luc Cracowski

**Affiliations:** 1 Clinical Pharmacology Unit, Inserm CIC03, Grenoble University Hospital, Grenoble, France; 2 Laboratoire HP2 - U1042, Inserm, Grenoble, France; 3 Joseph Fourier University, Grenoble, France; 4 Vascular Medicine Department, Grenoble University Hospital, Grenoble, France; 5 TIMC UMRCNRS 5525, Grenoble, France; Universidade Federal do Rio de Janeiro, Brazil

## Abstract

**Introduction:**

The treatment of scleroderma-related digital ulcers is challenging. The oral endothelin receptor antagonist (ERA) bosentan has been approved but it may induce liver toxicity. The objective of this study was to test whether ERAs bosentan and sitaxentan could be locally delivered using iontophoresis.

**Methods:**

Cathodal and anodal iontophoresis of bosentan and sitaxentan were performed on anaesthetized rat hindquarters without and during endothelin-1 infusion. Skin blood flow was quantified using laser-Doppler imaging and cutaneous tolerability was assessed. Iontophoresis of sitaxentan (20 min, 20 or 100 µA) was subsequently performed on the forearm skin of healthy men (n = 5).

**Results:**

In rats neither bosentan nor sitaxentan increased skin blood flux compared to NaCl. When simultaneously infusing endothelin-1, cathodal iontophoresis of sitaxentan increased skin blood flux compared to NaCl (AUC_0–20_ were 44032.2±12277 and 14957.5±23818.8 %BL.s, respectively; P = 0.01). In humans, sitaxentan did not significantly increase skin blood flux as compared to NaCl. Iontophoresis of ERAs was well tolerated both in animals and humans.

**Conclusions:**

This study shows that cathodal iontophoresis of sitaxentan but not bosentan partially reverses endothelin-induced skin vasoconstriction in rats, suggesting that sitaxentan diffuses into the dermis. However, sitaxentan does not influence basal skin microvascular tone in rats or in humans.

## Introduction

Systemic sclerosis (SSc) is a rare disease affecting digital microcirculation, leading to finger ulcers and in some cases to amputation [1]. Therapy of SSc-related ulcers is challenging. Bosentan, a non specific endothelin receptor antagonist (ERA), has been indicated to prevent digital ulcers in patients at risk, but it has no efficacy on existing ulcers [2]. Elevated aminotransferase levels is the main adverse effect of bosentan, with an annual rate of 10.1%, leading to therapy discontinuation in 3.2% of bosentan-naive patients [3]. Prostacyclin (PGI_2_) analogues are used intravenously [4], but their therapeutic effect is counterbalanced by potentially serious vasodilatation-induced side effects (e.g. severe headaches, flushing, tachycardia and hypotension).

The topical administration of these drugs may be a way of avoiding the toxicity of systemic treatments. Iontophoresis is a simple, non-invasive transdermal drug delivery method using a low-intensity electric current [5]. Some authors have highlighted the potential interest of iontophoresis of vasodilating drugs as a treatment for digital ulcers in SSc [6], [7] and previous work from our laboratory has suggested that PGI_2_ analogues are appropriate candidates [8]. Iontophoresis of ERAs could also be interesting but, to our knowledge, it has never been tested either in animals or in humans.

We conducted a laboratory and a clinical study to address this question. The main objective of the animal study was to assess whether iontophoretically-administered ERAs, bosentan and sitaxentan, increase cutaneous blood flux in rats. As a secondary objective, we tested the toxicity of the iontophoresis of ERAs. In a second study, we tested the effect of the iontophoresis of sitaxentan on human skin blood flux as well as cutaneous and systemic tolerability.

## Materials and Methods

### 1. Animal Study

#### Animals

Thirty-two male Wistar rats (eight-weeks old, 295–380 g; CERJ, Le Genest-St-Isle, France) were housed in controlled conditions conforming to the current French legislation and provided with standard rat chow. The protocol was approved by the Rhone Alpes Region Animal Ethics Committee (number 309). Rats were kept in a day/night cycle of 12 h/12 h with food and water at will. Preparation of the animals for iontophoresis has been previously described [8].

#### Drugs

Bosentan sodium salt (Actelion Pharmaceuticals, Allschwil, Switzerland) (MW 573.6 g.mol^−1^) and sitaxentan sodium salt (Pfizer Inc, Groton, CT, USA) (MW 476.9 g.mol^−1^) were used for iontophoresis, and isotonic sodium chloride (NaCl 0.9%) (Aguettant, Lyon, France) was used as a control. Solutions were prepared extemporaneously by diluting 20 mg of bosentan or sitaxentan in 3.6 mL and 4.4 mL of NaCl 0.9%, respectively, to obtain 10^−2^ M solutions. These solutions were subsequently diluted with NaCl 0.9% to obtain 10^−3^ and 10^−4^ M solutions. The pH of all solutions was determined before iontophoresis using a microprocessor-based pH meter (pH 210, Hanna Instruments, Woonsocket, RI, USA). The solutions were pH 5.5 to 6.5, which is suitable for epidermal application. Endothelin-1 (Sigma-Aldrich, Saint-Quentin Fallavier, France) (MW 2491.9 g.mol^−1^) was diluted in water for injection to obtain 0.5 10^−5^ M, 0.5 10^−6^ M, 0.5 10^−7^ M, 0.5 10^−8^ M and 0.5 10^−9^ M solutions. In order to assess the concentration of endothelin needed to decrease skin blood flux, dose-response was assessed with 0.5 10^−9^ to 0.5 10^−5^ M solutions (n = 3). Only endothelin at 0.5 10^−5^ M decreased skin blood flux. This concentration was then used throughout the study.

**Figure 1:**
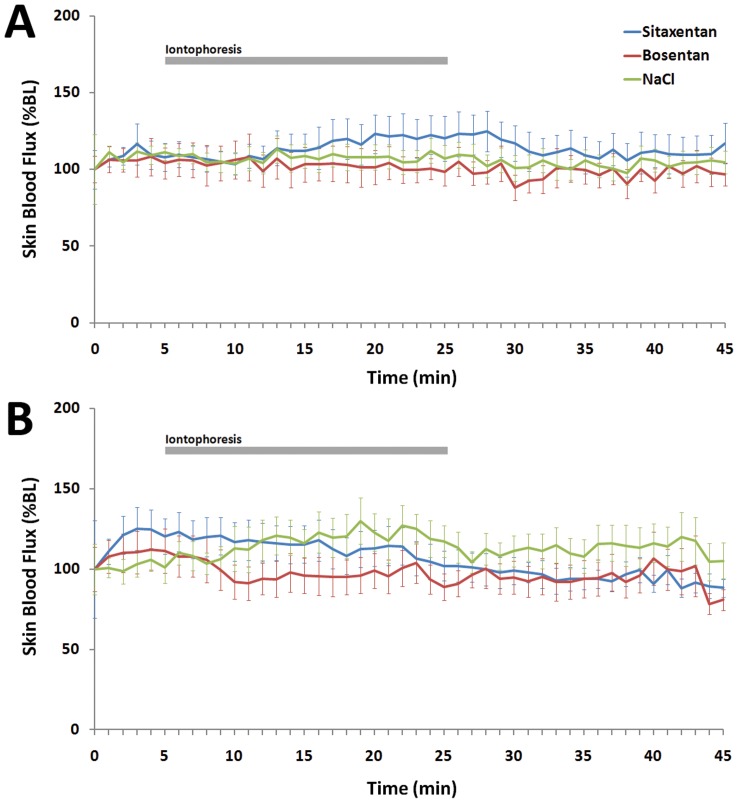
Effect of iontophoresis of bosentan 10 ^−**2**^ **M, sitaxentan 10** ^−**2**^ **M and NaCl 0.9 % on skin blood flux in rats, expressed as mean (± SEM) percentage change from baseline (%BL), in the cathodal direction (Fig. 1A) and the anodal direction (Fig. 2B).**

**Table 1:** Effect of cathodal and anodal iontophoresis of bosentan and sitaxentan on skin blood flux recorded on the back and the hind legs of rats.

Polarity		NaCl	Bosentan	P-value*	Sitaxentan	P-value*
Cathodal (n = 9)	AUC_0–20_	9598±23693	5061±39161	0.44	13553±33368	0.86
	AUC_0–40_	15142±48007	2717±64048	0.44	32534±74077	0.51
Anodal (n = 9)	AUC_0–20_	15123±26678	1090±40367	0.24	20851±39909	0.80
	AUC_0–40_	32436±55790	4215±71472	0.11	20286±64462	0.51

Data are expressed as area under the curve of the percentage change from baseline (expressed as %BL.s) during the 20-min iontophoresis (AUC_0–20_) and during the whole recording (AUC_0–40_).

* vs NaCl (Wilcoxon rank test).

#### Laboratory experimental procedures

The rats were anesthetized with sodium pentobarbital (50 mg.kg^−1^ i.p.) and were maintained in the prone position for the duration of the whole experiment, with the back uppermost. Experiments were performed in a temperature controlled room, and the rats were placed on a thermal pad, the temperature being maintained at 37.5°C (Harvard apparatus).

*Experiment 1:* Iontophoresis of bosentan, sitaxentan and NaCl (20 min, 100 µA) were simultaneously performed in the same animal using cathodal or anodal current (n = 9 for each series). Mean arterial blood pressure was measured by plethysmography using the tail cuff method, before and immediately after iontophoresis. Before iontophoresis each rat was inspected to ensure that the hairless skin in the back and the hind legs was intact. Photographs were taken before iontophoresis, immediately after and 3 days later. A cutaneous score was used to assess skin tolerability, based on the International Contact Dermatitis Research Group scoring [9]. Negative reactions were coded grade 0; weak reactions (grade 1) are characterized by non-vesicular erythema. Strong positive reactions (grade 2) are characterized by erythema associated with vesicles. Extreme positive reactions (grade 3) are bullous reactions. Irritant reactions (that we coded grade 4) are characterized by necrosis.

*Experiment 2:* Catheters were inserted in both carotid arteries. Arterial pressure (Powerlab, ADInstrument) was recorded through the first catheter and endothelin (5 nmol.kg^−1^ i.a. bolus followed by 0.5 µL.min^−1^ infusion) was administered through the second catheter. We chose i.a. rather than i.v. endothelin infusion due to its removal in the pulmonary circulation [10]. Moreover, prolonged endothelin infusion was not possible through the catheter used to record arterial pressure. This invasive procedure led to significant hemodynamic instability in four animals in which iontophoresis was not performed. Iontophoresis of bosentan, sitaxentan and NaCl (20 min, 100 µA) were then simultaneously performed in the same animal using cathodal (n = 8) or anodal (n = 6) currents. Histopathologic examination of full-thickness skin biopsies from bosentan, sitaxentan, NaCl treated areas and from one non-treated skin area was realized 15 min after the end of iontophoresis (n = 9∶5 after cathodal and 4 after anodal iontophoresis). The thirty-six biopsies were fixed in AFA fluid (5% acetic acid, 75% absolute ethyl alcohol, and 18% water; Carlo Erba) paraffin-embedded and stained with hematoxylin, eosin, and safran. In order to evaluate the effect of the treatment on the skin, various features were sought in the different skin layers [the histopathologic examination procedure has been previously described [8]].

#### Skin blood flux measurement and data analysis

Three 1.2 cm^2^ circular iontophoresis electrodes (PeriIont System, Perimed, Järfälla, Sweden) containing bosentan, sitaxentan or NaCl were placed on the hairless skin of the lower back/hind legs. Passive electrodes were placed on the back of the neck, as previously described [8]. Skin blood flux was continuously recorded with laser Doppler imaging (LDI; PeriScan PIM 3, Perimed, Järfälla, Sweden). The laser head was placed 20 cm above the skin. The resolution was 2 mm step length and LDI scans were taken every minute.

In experiment 1, iontophoresis was started after a 5-min baseline (BL) recording. Skin blood flux was then recorded during iontophoresis and during the 20 min following the end of iontophoresis, and expressed as arbitrary perfusion units (PU). In order to take into account inter-individual BL variations, data were subsequently expressed as a percentage change from BL (%BL). Then, a minute by minute analysis was performed to calculate the area under the curve during iontophoresis (AUC_0–20_, in %BL.s) and during the whole recording (AUC_0–40_).

In experiment 2, the skin blood flux was recorded for 5-min under resting conditions. Iontophoresis was performed 5 min after endothelin infusion started, time for blood flux to decrease and reach a plateau (baseline). Data were expressed as percentage change between baseline and the iontophoresis plateau (averaged over 10 min) (expressed as %BL). Then, AUC_PU__0–20_ was calculated as described above and expressed as %BL.s. In order to take into account endothelin-induced variations in blood pressure, we expressed data as cutaneous vascular conductance (CVC), i.e. flux divided by arterial pressure averaged over 1 min (in mV.mm Hg^−1^) [11], [12], to subsequently calculate AUC_CVC 0–20_ (expressed as %BL.s).

**Figure 2:**
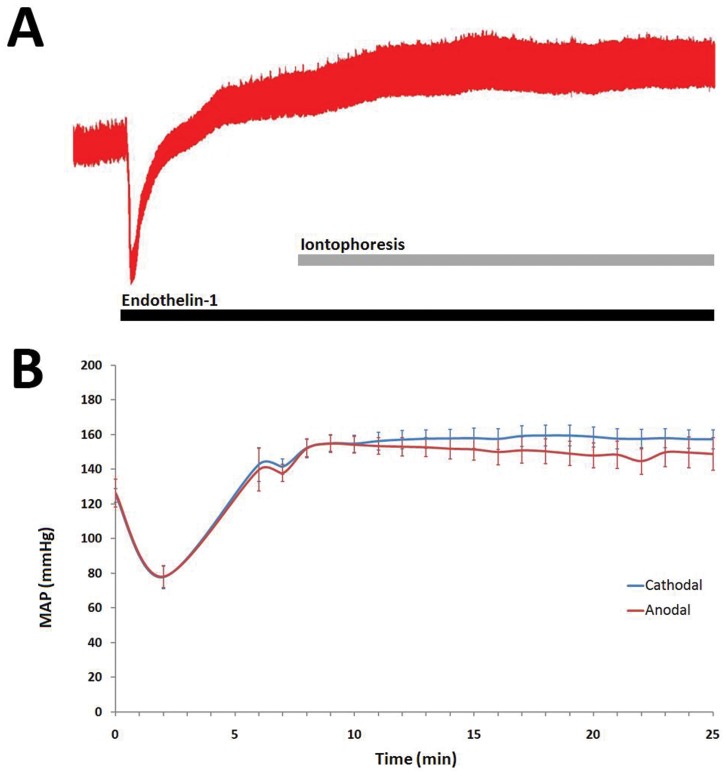
Typical tracing of the effect of i.a. endothelin 0.5 10^−5^ M on arterial pressure in rats: the initial transient decrease of arterial pressure is followed by a sustained increase (Fig 2A). Mean (± SEM) arterial pressure during i.a. endothelin perfusion and iontophoresis of bosentan or sitaxentan in the cathodal (blue line) and anodal (red line) directions in rats (Fig. 2B).

**Table 2:** Effect of cathodal and anodal iontophoresis of bosentan and sitaxentan on skin blood flux recorded on the back and the hind legs of rats during endothelin infusion.

		NaCl	Bosentan	P-value*	Sitaxentan	P-value*
Cathodal (n = 8)	%BL	8.21±20	19.3±23.1	0.4	34.3±15.5	0.02
	AUC_PU 0–20_	14957±23818	27569±25533	0.26	44032±12277	0.01
	AUC_CVC 0–20_	3687±26073	12370±24147	0.33	27835±18050	0.01
Anodal (n = 6)	%BL	18.7±24.4	21.4±26.2	0.75	26.4±24.2	0.92
	AUC_PU 0–20_	29513±34899	31898±30426	0.92	24747±28687	0.6
	AUC_CVC 0–20_	19792±37485	19063±16564	0.75	13405±34335	0.75

Data are expressed as percentage change between baseline and iontophoresis plateau (%BL), and as the area under the curve (AUC_0–20_) of the percentage change from baseline (expressed as %BL.s), whether calculated from arbitrary perfusion units (PU) or from cutaneous vascular conductance (CVC) in order to take into account variations in arterial blood pressure.

* vs NaCl (Wilcoxon rank test)**.**

P-values <0.025 were considered as significant.

**Figure 3:**
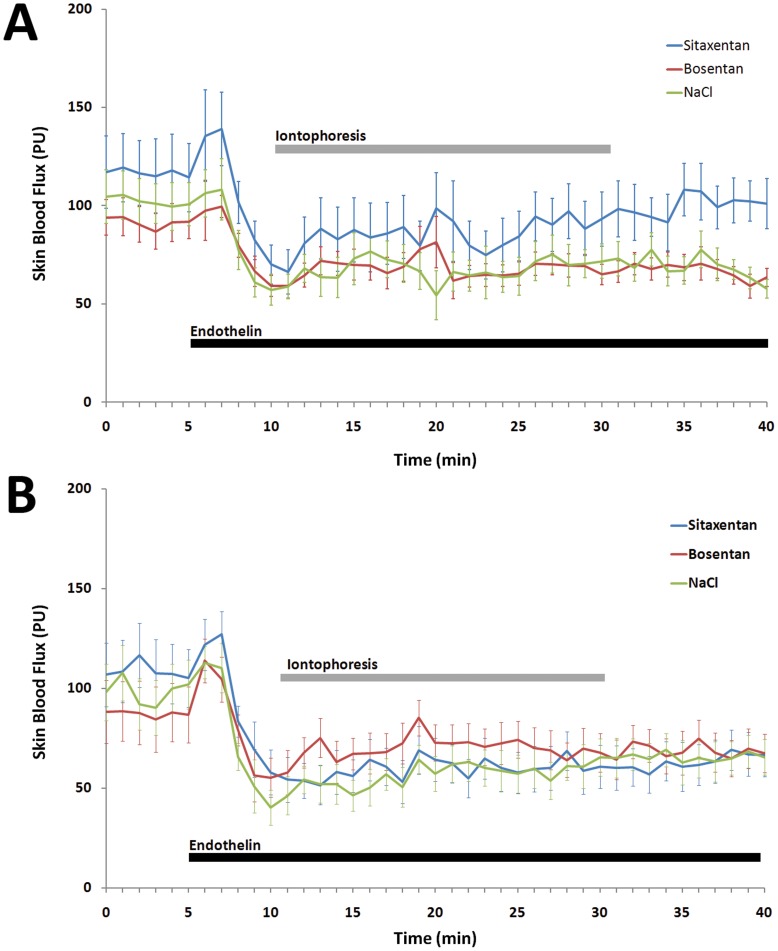
Effect of iontophoresis of bosentan 10 ^−**2**^ **M, sitaxentan 10** ^−**2**^ **M and NaCl 0.9% on skin blood flux during infusion of i.a. endothelin 0.5 10** ^−**5**^ **M in rats, expressed as arbitrary perfusion units mean (mean±SEM), in the cathodal direction (** **Fig 3A** **) and anodal direction (Fig. 3B).**

### 2. Clinical Study

#### Study population

We recruited healthy men through the Clinical Research Centre database. Inclusion criteria were an age of 18–65 years with no significant medical history. Non-inclusion criteria included any allergies to local anaesthetics, cigarette smoking or dermatologic disease on the arm or forearm. Any abnormal liver enzyme level was also an exclusion criterion. Grenoble Institutional Review Board (IRB n°6705) approval was obtained in September 2009 and each volunteer gave written informed consent before participation.

#### Study design

This was an open-label pharmacology study. Upon arrival at the centre volunteers were placed in a temperature-controlled room (23+/−1°C) during 1 hour for acclimatization. They remained supine for the whole experiment.

Three consecutive visits were planned for each volunteer, separated by 7 days +/− 3. Visit 1 was the initial enrolment visit. During visit 2, iontophoresis of sitaxentan 10^−2^ M and NaCl 0.9% were performed. Volunteers arrived fasted for at least 6 hours and were allowed to eat only after the end of the experiment. Visit 3 was a safety monitoring clinical visit. Skin photographs were taken before the start of iontophoresis and immediately after iontophoresis (visit 2), and 7 days later (visit 3). Liver enzyme levels were assessed at visits 1 and 3.

**Figure 4:**
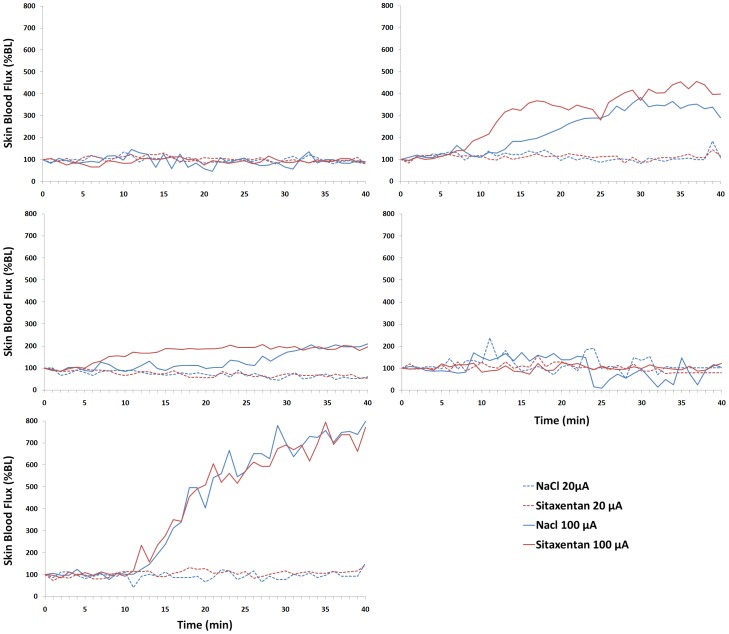
Effect of iontophoresis of sitaxentan 10 ^−**2**^ **M (red lines) and NaCl 0.9 % (blue lines) on skin blood flux, expressed as a percentage change from baseline (%BL), in the forearm skin of five healthy volunteers.** Current intensity was set at 20 µA (dashed lines) or 100 µA (solid lines).

#### Iontophoresis protocol and skin blood flux measurement

Four iontophoresis skin sites were randomly chosen on the ventral side of the right forearm, more than 5 cm from the elbow and the prominence of the wrist, avoiding visible veins. Two of them were pre-treated with 1 g of lidocaine/prilocaine cream (5 g tubes containing 125 mg lidocaine and 125 mg prilocaine, Anesderm®, Pierre Fabre, Boulogne, France) in order to attenuate axon reflex-induced hyperaemia [13]. An occlusive transparent dressing was placed over the cream to enhance cutaneous diffusion. Each anesthetized area was larger than the area of the iontophoresis chambers.

One hour after lidocaine/prilocaine application (corresponding to the acclimatization period) the cream was removed with a cotton swab. The two pre-treated skin sites were then equipped with 1.2 cm^2^ circular iontophoresis chambers (PeriIont System, Perimed, Järfälla, Sweden) containing sitaxentan or NaCl. After a 5-min baseline recording, cathodal iontophoresis was simultaneously performed for 20 minutes with a current intensity set at 20 µA. After iontophoresis, skin blood flux was recorded for an additional 20 min. During the experiment, the two remaining skin sites were pre-treated with local anaesthetic cream for one hour. The same protocol as described above was then performed on these skin sites, the only difference being the current intensity set at 100 µA.

Skin blood flux was recorded throughout with laser-Doppler imaging (PeriScan PIM 3, Perimed, Järfälla, Sweden). The resolution was 1 mm step length and LDI scans were taken every 30 seconds. Before recording, the arm was immobilized with a vacuum cushion to decrease artefacts related to arm movement. Baseline and maximal skin blood flux were averaged over 5 min. Data were subsequently expressed as a percentage change from BL (%BL) and AUC_0–40_ were calculated (expressed as %BL.s).

### 3. Statistical Analysis

Categorical data were reported as frequency and percentage and continuous data as mean and standard deviation. Two-sided nonparametric tests were used throughout (Friedman test, and Wilcoxon test for paired comparisons). We considered p values <0.05 as significant, corrected by Bonferroni’s method for multiple comparisons. Statistical analysis was performed with SPSS 13.0 for Windows (SPSS Inc, Chicago IL, USA).

## Results

### 1. Animal Study

#### Effect of iontophoresis of bosentan and sitaxentan on skin blood flux

Neither cathodal nor anodal iontophoresis of bosentan 10^–2^ M or sitaxentan 10^−2^ M induced any significant change in skin blood flux compared to NaCl (Table 1; Figure 1). Iontophoresis did not significantly affect blood pressure (87.5±17.9 mmHg before and 90.6±10.2 mmHg after iontophoresis; P = 0.7). Similarly, we observed no significant effect of bosentan or sitaxentan at 10^−3^ M or 10^−4^ M (data not shown for clarity).

#### Effect of cathodal and anodal iontophoresis of bosentan and sitaxentan after administration of endothelin

Resting skin blood flux before cathodal iontophoresis at bosentan, sitaxentan and NaCl skin areas were 91.5±25 PU, 116.7±48.8 PU and 102.2±32.1 PU, respectively (P = 0.2). Endothelin decreased skin blood flux to 57.8±17.4 PU, 65.1±31.6 PU and 59.4±17.7 PU, respectively (P = 0.7). We observed an initial transient decrease in blood pressure immediately after endothelin infusion was started, followed by a sustained increase (Figure 2). Cathodal iontophoresis of sitaxentan significantly increased skin blood flux compared to NaCl. However, we observed no significant difference between bosentan and NaCl (Table 2; Figure 3A).

Resting skin blood flux before anodal iontophoresis for bosentan, sitaxentan and NaCl skin areas were 88.3±16.1 PU, 106.9±39.1 PU and 98.1±21.7 PU, respectively (P = 0.3). Endothelin infusion decreased skin blood flux to 59.7±33.8 PU, 55.1±26.5 PU and 47.5±18.8 PU, respectively (P = 0.5). Anodal iontophoresis of bosentan or sitaxentan did not significantly increase skin blood flux compared to NaCl (Table 2; Figure 3B).

#### Skin tolerability of the iontophoresis of bosentan and sitaxentan

No skin side-effects were observed. In experiment 1, all the cutaneous scores were 0 both immediately after the procedure and at day 3. In experiment 2, no abnormal histopathologic features (e.g. perivascular inflammatory infiltration in the papillary dermis; dissociation of keratinocytes due to epidermal edema; migration of inflammatory cells from the dermis to the epidermis) were found in any of the skin biopsies.

### 2. Clinical Study

#### Study population

Six male volunteers were enrolled in the study. One of them did not attend visits 2 and 3 and was excluded. The characteristics of the population were: mean age 22.8±1.9 years and body mass index 22.1±1.5 kg/m^2^. Mean systolic/diastolic arterial pressure at inclusion was 115±7.4 / 77±10.9 mm Hg.

#### Effect of cathodal iontophoresis of sitaxentan on skin blood flux

Neither sitaxentan nor NaCl increased skin blood flux when the iontophoresis current intensity was 20 µA (AUC_0–40_ were −2785±35811 %BL.s and −3315±42523 %BL.s, respectively; P = 0.69). Cathodal iontophoresis of sitaxentan induced an inconsistant increase in skin blood flux when the current intensity was set at 100 µA (AUC_0–40_ was 289409±348171 %BL.s). However, a similar increase was observed with NaCl (AUC_0–40_ was 236430±348333 %BL.s; P = 0.22). Individual tracings show the high heterogeneity in the response to iontophoresis (Figure 4).

#### Tolerability

No side-effect occurred. We observed no significant change in mean arterial pressure during iontophoresis of sitaxentan. No squamous, vesicular or bullous lesions were observed at the end of the experiment or at the safety visit. Erythema was observed in one participant. It was localized to the area surrounding the anesthetized area and was attributed to hypersensitivity to the adhesive of the dressing. Aspartate transaminase (AST)/alanine transaminase (ALT) at visit 1 and visit 3 for each volunteer were: 34/17 and 36/14 UI.L^−1^, 18/27 and 21/21 UI.L^−1^; 16/15 and 20/12 UI.L^−1^; 30/25 and 31/25 UI.L^−1^; 30/18 and 26/29 UI.L^−1^.

## Discussion

This study showed that in basal conditions iontophoresis of bosentan (in rats) or sitaxentan (in rats and humans) did not increase skin blood flux. However, after endothelin infusion, iontophoresis of sitaxentan significantly increased cutaneous blood flux in rats, suggesting that the absence of effect initially observed was due to low endothelin-dependent basal skin microvascular tone rather than the absence of diffusion of the antagonist into the skin. However, no effect was seen with bosentan. Iontophoresis of ERAs was well tolerated both in animals and humans.

Iontophoretic transport is influenced by several parameters, including drug concentration, molecular weight, ionization and solution pH or current strength and skin hydration and resistance [14]. Bosentan and sitaxentan are small molecules with isoelectric points (pI) at 2.64 and 4.57, respectively. These chemical properties make these ERAs appropriate candidates for cathodal iontophoresis, being negatively charged at neutral pH. However, in basal conditions, we were not able to see any effect of the iontophoresis of bosentan or sitaxentan on skin blood flux in rats (experiment 1). In order to test whether the absence of effect was related to low endothelin-dependent vascular tone in the skin of healthy rats or to the absence of iontophoretic transport into the dermis, we repeated the experiment while infusing endothelin (experiment 2). The results of this series suggest that the cathodal iontophoresis of sitaxentan enabled the drug to be locally administered in sufficient concentration to partially reverse the sustained effect of endothelin on the skin microcirculation. However, such an effect was not observed with bosentan, suggesting that we were unable to reach sufficient skin concentration to reverse endothelin vasoconstriction. It is of interest that we obtained similar results when data were expressed as cutaneous vascular conductance, to take into account the effect of endothelin on arterial blood pressure. The effect of endothelin on blood pressure was comparable to that previously described: an initial transient decrease in blood pressure immediately after endothelin infusion was started was followed by a prolonged increase [10].

The relationship between the pharmacodynamic effect of ERAs on microvascular function and their efficacy in the prevention of digital ulcers in patients at risk remains uncertain. Two groups have studied the effect of the systemic administration of bosentan on skin blood flux in patients with SSc [15], [16]. Hettema *et al* did not show any difference in blood flux during acetylcholine and sodium nitroprusside iontophoresis, used as pharmacological tests of microvascular endothelium-dependent and endothelium-independent function, respectively [17]. Conversely, Rosato *et al* showed an increase in skin perfusion 8 weeks after bosentan therapy was started in SSc patients with SSc and pulmonary hypertension. In SSc patients without pulmonary hypertension however, there was no significant difference [15]. These discrepancies could be due to higher endothelin-dependent vascular tone in patients with pulmonary hypertension.

Insufficient diffusion of ERAs into the skin after oral administration is another hypothesis. Indeed, when administered directly into the dermis BQ-123 (an ET_A_ receptor antagonist) significantly increased skin blood flux in healthy volunteers [18]. Interestingly, intradermal BQ-788 (an ET_B_ receptor antagonist) also increased blood flow [18], suggesting that both ET_A_ and ET_B_ contribute to endothelin-mediated basal microvascular tone in the human skin. On the other hand, although both ET_A_ and ET_B_ mediate vasoconstriction in rat skin [19], injection of BQ-123 and bosentan (non specific ET_A_ and ET_B_ antagonist) did not alter basal skin blood flow in rats but reversed the effects of endothelin [20], which is consistent with our findings.

Therefore, iontophoresis probably did not allow sufficient concentrations of ERAs to be reached in human skin to be able to show any effect on the microvasculature. We did not use intradermal injections or microdialysis as these methods would be too far removed from the initial therapeutic objective, i.e. iontophoresis in physiological conditions.

In healthy participants, we did not observe any increase in skin blood flux for either sitaxentan or NaCl when the current intensity was 20 µA (as previously described [13]), whereas it was inconsistent at 100 µA. Indeed, one of the main issues when performing iontophoresis is the non specific effect of the current itself, often referred to as “current-induced vasodilation”. The amplitude of this vasodilation depends on the delivered electrical charge (i.e. the product of current intensity by duration of application) [21], which may explain the discrepancy. As the neural axon reflex has been suggested to be involved in current-induced vasodilation [21], we pre-treated the skin sites with a local anaesthetic (lidocaine/prilocaine). However, this did not abolish current-induced vasodilation in all subjects when the current intensity was 100 µA, suggesting the involvement of pathways other than sensory nerve-dependent mechanisms.

In humans, individual data showed comparable skin blood flow for sitaxentan and NaCl in each participant (individual data not shown for clarity). The highly variability observed at 100 µA is due to major current-induced vasodilation in two participants (at all sites), whereas in the three other subjects we only observed a weak or even no effect at all. This difference could be due to inter-individual differences in skin resistance, which was not recorded at this early stage. Nonetheless, it is unlikely to have a major impact on the conclusion of these experiments as each subject was his own control. Indeed, the iontophoresis were simultaneously performed at both sites with the same distance between electrodes.

The clinical study was prematurely stopped after the manufacturer decided to discontinue all ongoing clinical trials with sitaxentan and to withdraw the commercial drug, because of several cases of fatal liver injury [22]. Nonetheless, despite the small sample size, there was no effect of iontophoresis of sitaxentan at 20 µA in any of the five participants, whereas in our experience with other molecules this current intensity leads to sustained vasodilation in humans [23]. There was an inconsistant effect at 100 µA, which is more difficult to interpret considering the small sample size and the large variability of the response. Altogether, and considering the elevated concentration used in the clinical study, our data suggest that iontophoresis is not an appropriate route of administration for sitaxentan.

Of note, we observed a difference (although not significant) in the response to NaCl between cathodal and anodal iontophoresis, the response being more pronounced in the anodal direction. This might be surprising as Durand previously showed that current-induced vasodilation was higher with cathodal iontophoresis [21]. Nonetheless, several differences should be pointed out: Durand used tap water whereas we used NaCl (which is known to make a significant difference [24]); their study was in humans, ours was in rats; they showed a marked difference, ours is modest considering the variability and does not reach significance.

It should be noted that the iontophoresis of ERAs was well tolerated both in animals and humans. No change in arterial blood pressure was observed and skin tolerance was excellent, although the skin biopsies only permitted us to detect immediate skin toxicity. Finally, it did not induce any significant increase in aspartate transaminase or alanine transaminase in humans.

In conclusion, this study shows that cathodal iontophoresis of sitaxentan but not bosentan partially reverses endothelin-induced skin vasoconstriction in rats, suggesting that sitaxentan diffuses into the dermis. Nevertheless, although the clinical study with sitaxentan had to be interrupted, we did not observe any effect of iontophoresis at 20 µA on skin blood flow in humans, and no clear-cut effect at 100 µA. Iontophoresis of other ERAs such as ambrisentan or macitentan could be an interesting alternative that remains to be explored.
